# Preventive Effect of Shenfu Injection on Arrhythmia After Percutaneous Coronary Intervention in Patients With ST-Segment Elevation Myocardial Infarction: A Prospective Randomized Controlled Trial

**DOI:** 10.1155/cdr/4097327

**Published:** 2025-11-30

**Authors:** Xiaohan Qiu, Yue Wang, Jiahan Ke, Min Wang, Huasu Zeng, Huafang Zhu, Jun Gu

**Affiliations:** ^1^Department of Cardiology, Shanghai Ninth People's Hospital, Shanghai Jiaotong University School of Medicine, Shanghai, China; ^2^Shanghai Huangpu District Geriatric Care Hospital, Shanghai, China

**Keywords:** randomized controlled trial, Shenfu injection, ST-segment elevation myocardial infarction, traditional Chinese medicine

## Abstract

**Background and Aims:**

Arrhythmias and major adverse cardiac events remain significant complications following ST-segment elevation myocardial infarction (STEMI). Shenfu injection, a traditional Chinese medicine formulation, has shown cardioprotective effects in preclinical studies. This trial is aimed at investigating whether Shenfu injection as an adjunctive therapy to standard treatment could reduce arrhythmias and improve clinical outcomes in patients with STEMI undergoing percutaneous coronary intervention (PCI).

**Methods:**

A single-center, prospective, randomized, controlled trial was conducted at Shanghai Ninth People's Hospital among 245 patients with STEMI undergoing PCI. Participants were randomized to receive either standard therapy plus Shenfu injection (50 mL, administered intravenously twice daily for five consecutive days) (*n* = 123) or standard therapy alone (*n* = 122). The primary endpoint was the incidence of in-hospital arrhythmias. Secondary endpoints included major adverse cardiac events (MACEs) during the 12-month follow-up period and cardiac magnetic resonance imaging parameters.

**Results:**

A total of 245 patients underwent randomization (123 assigned to Shenfu injection group and 122 assigned to control group). During hospitalization, patients assigned to Shenfu injection had a significantly lower incidence of arrhythmias compared with the control group (24.4% vs. 38.5%, *p* = 0.017), with the most pronounced effect on frequent ventricular premature contractions (7.3% vs. 15.5%, *p* = 0.042). After adjustment for key baseline covariates including age, coronary artery disease extent, myocardial injury markers (CK-MB max and TNI max), left ventricular ejection fraction, B-type natriuretic peptide, door-to-balloon time, hypertension, and diabetes mellitus, Shenfu injection remained independently associated with reduced risk of in-hospital arrhythmias (adjusted OR 0.454, 95% CI: 0.249–0.827, *p* = 0.010). Cardiac magnetic resonance imaging performed in 174 patients revealed significantly smaller infarct size (16.1 ± 9.1 vs. 20.8 ± 13.1 g, *p* = 0.007) and lower incidence of microvascular obstruction (45.0% vs. 65.0%, *p* = 0.008) in the Shenfu group, with both parameters showing significant positive correlations with arrhythmia occurrence. During the 12-month follow-up, patients receiving Shenfu injection had a higher event-free rate from MACEs compared with the control group (12-month Kaplan-Meier event-free rate estimates, 84.6% vs. 73.0%, respectively; *p* = 0.028).

**Conclusions:**

Treatment with Shenfu injection as an adjunctive therapy to standard treatment in patients with STEMI undergoing PCI significantly reduced in-hospital arrhythmias, infarct size, microvascular obstruction, and major adverse cardiac events during a 12-month follow-up period. The independent effect on arrhythmias after comprehensive statistical adjustment and the demonstrated correlation between reduced myocardial damage and arrhythmia prevention suggest the potential therapeutic value of Shenfu injection in improving both short-term and long-term outcomes in STEMI patients.

**Trial Registration:**

Chinese Clinical Trial Registry Identifier: ChiCTR2200066918

## 1. Introduction

Arrhythmia is the most common and serious complication in patients with STEMI, with an incidence rate of 70%–80%, and malignant arrhythmia remains the leading cause of early mortality in STEMI [1–3]. STEMI-induced pathological changes include not only myocardial fibrosis but also electrophysiological remodeling in surviving cardiomyocytes at the infarct border, characterized by reduced K^+^ influx during repolarization, decreased connexin 43 expression, and disturbed intracellular Ca^2+^ homeostasis [4–6]. The incidence of post-PCI arrhythmias has significantly decreased with the advancement of PCI. Winkler et al. conducted a subgroup analysis of the IMMEDIATE AIM study, revealing that among 278 ACS patients, the incidence of ventricular premature beats within 24 h of admission was 22%, with malignant arrhythmias occurring in only 1% [7]. Nevertheless, both tachyarrhythmias and bradyarrhythmias following STEMI remain independent predictors of adverse cardiovascular events during hospitalization and long-term follow-up [8].

Current therapeutic strategies for STEMI-associated arrhythmias primarily rely on pharmacological interventions (such as amiodarone or lidocaine for ventricular tachyarrhythmias) or device therapy (temporary pacing for severe bradyarrhythmias) [9–11]. However, these approaches have significant limitations, including adverse drug reactions and procedural complications. To date, no preventive strategies have demonstrated clear efficacy in reducing post-PCI arrhythmias in STEMI patients.

Shenfu injection, derived from Panax ginseng and *Aconitum carmichaelii*, is a traditional Chinese medicine preparation that shows promising effects in cardiovascular medicine [12, 13]. This extraordinary formulation demonstrates powerful efficacy against life-threatening conditions including cardiogenic shock, persistent heart failure, and complex arrhythmias through its unique bidirectional heart rate regulation—an intelligent mechanism that adapts to cardiac needs like a masterful conductor [12, 14, 15]. Operating through sophisticated pathways, Shenfu enhances sinoatrial node function via norscopolamine mechanisms, dramatically improves myocardial contractility while optimizing coronary blood flow, and deploys an elegant multitargeted defense against ischemia–reperfusion injury through potent antioxidant actions, precise calcium homeostasis restoration [16–19], and strategic inflammatory response modulation [19]—representing a groundbreaking fusion of ancient wisdom and cutting-edge science that offers renewed hope for millions suffering from heart disease worldwide.

Previous experimental studies demonstrated that Shenfu injection improved myocardial metabolism in ventricular fibrillation models and reduced ischemia–reperfusion injury [20–22]. A pilot clinical study suggested its potential in reducing infarct size in STEMI patients undergoing PCI, though larger trials are needed for validation. Additionally, Shenfu injection has been shown to improve heart rate variability in post-PCI STEMI patients. Our preliminary retrospective analysis indicated that Shenfu injection might reduce in-hospital arrhythmia rates in STEMI patients. Based on these findings, we designed this single-center, prospective, randomized controlled trial to evaluate whether Shenfu injection reduces the incidence of post-PCI arrhythmias and improves clinical outcomes in STEMI patients. The results of this study will provide crucial evidence for establishing standardized protocols for Shenfu injection in modern cardiovascular care.

## 2. Method

### 2.1. Trial Design

This was a single-center, prospective, randomized, controlled trial conducted at Shanghai Ninth People's Hospital, Shanghai Jiaotong University School of Medicine. The trial was conducted in accordance with ethical standards and was approved by the Institutional Review Board of Shanghai Ninth People's Hospital, Shanghai Jiaotong University School of Medicine (Approval Number: SH9H-2022-T240-2, approved on October 24, 2022). Written informed consent was obtained from all participants prior to inclusion.

### 2.2. Trial Participants

Patients with STEMI who underwent primary PCI were enrolled. The diagnosis of STEMI was based on the 2025 ACC/AHA guidelines [23], defined as dynamic changes in cardiac injury biomarkers (preferably cardiac troponin with at least one value exceeding the 99th percentile upper reference limit) with at least one of the following: persistent symptoms of myocardial ischemia (chest tightness, chest pain, difficulty breathing, etc.), ischemic ECG changes (ST-segment elevation, pathological Q waves, or new left bundle branch block), or imaging evidence showing new loss of viable myocardium or new regional wall motion abnormality.

### 2.3. Inclusion and Exclusion Criteria

**Figure 1:**
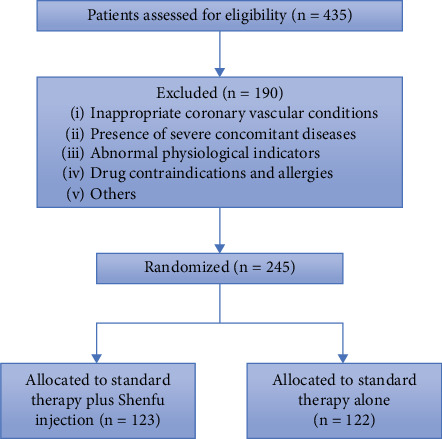
Study flow diagram.

A total of 435 patients were assessed for eligibility. Inclusion criteria were (1) patients aged 18–80 years with STEMI, (2) patients undergoing primary PCI, and (3) patients who understood the purpose of the study and voluntarily provided written informed consent. Exclusion criteria included (1) left main coronary artery disease, graft vessel, or in-stent acute occlusion; (2) cardiogenic shock; (3) arrhythmias defined by the study already present at admission; (4) systolic blood pressure exceeding 160 mmHg; (5) concomitant severe diseases (such as malignant tumors, organ transplantation, or candidates); (6) known allergy to Shenfu injection; and (7) severe liver or kidney dysfunction. A total of 190 patients were excluded based on these criteria, and 245 patients were randomized into two groups: 123 patients allocated to standard therapy plus Shenfu injection and 122 patients allocated to standard therapy alone (Figure 1).

### 2.4. Intervention, Randomization, and Treatment

This was a single-center, open-label, nonblinded randomized controlled trial. Eligible patients were randomly assigned in a 1:1 ratio using a random number table to receive either standard therapy plus Shenfu injection (intervention group) or standard therapy alone (control group). The control group received guideline-recommended treatment including antiplatelet agents, lipid-lowering and plaque-stabilizing medications, and inhibitors of myocardial remodeling. The intervention group received standard therapy plus Shenfu injection (Huarun Sanjiu [Ya'an] Pharmaceutical Co., Ltd., Approval Number: NMPN Z51020664, specification: 10 mL∗5 vials/box). Shenfu injection is a traditional Chinese medicine preparation extracted from red ginseng and aconite root using modern pharmaceutical technology, presenting as a light yellow or light yellowish-brown clear liquid. According to component analysis studies, the injection contains ginsenosides (600–900 *μ*g/mL) with Rg1, Rb1, and Rc as major components, alkaloids (2–12 *μ*g/mL) with benzoylmesaconine (BMA) and fuziline (FN) as predominant compounds, along with carbohydrates, inorganic salts, and amino acids. The dosing regimen was 50 mL Shenfu injection diluted in 100 mL of normal saline, administered intravenously twice daily at an infusion rate of 40–60 drops/minute for five consecutive days.

### 2.5. Coronary Angiography and Percutaneous Coronary Intervention

For STEMI patients, primary PCI was performed immediately upon diagnosis. All PCI procedures followed the center's standard techniques. Anticoagulation was administered according to the center's standard protocol. Operators determined the appropriate stent size and length to ensure complete coverage from “normal to normal” segments (both ends of the lesion), with a stent to reference vessel diameter ratio of 1:1. The use of thrombus aspiration and predilation was at the operator's discretion.

### 2.6. Post-PCI Management Until Discharge

Patients were treated according to the hospital's standardized treatment protocol after the procedure. Medication therapy included aspirin, ticagrelor (or clopidogrel), low molecular weight heparin, statins, RAS inhibitors, beta-blockers, and so on. All patients were admitted to the coronary care unit (CCU) and underwent continuous cardiac monitoring immediately upon admission until discharge. Real-time cardiac monitoring was performed using bedside monitors with automatic arrhythmia alarm systems. All arrhythmic events were captured by automatic alarm systems and manually verified by trained research physicians. Postprocedural care also included sheath removal, electrocardiogram (immediately after 2 h postprocedure), monitoring of CK, CK-MB, cardiac ultrasound, and assessment of myocardial ischemia, angina, and other MACE events.

### 2.7. Cardiac Magnetic Resonance (CMR) Imaging

CMR imaging was performed in all subjects 5–7 days after the index procedure. Standard cine images were acquired to assess left ventricular volumes and function. Late gadolinium enhancement images were obtained to evaluate infarct size and the presence of microvascular obstruction (MVO). Left ventricular end-diastolic volume (LVEDV), left ventricular end-systolic volume (LVESV), left ventricular ejection fraction (LVEF), left ventricular mass, infarct size, and MVO were measured. Infarct size was quantified in grams and as a percentage of left ventricular mass. MVO was defined as the hypoenhanced region within the hyperenhanced infarcted myocardium. The third-party software CVI 42 (Circle Cardiovascular Imaging, Canada) was used to analyze the magnetic resonance images.

### 2.8. Follow-Up

All patients were followed up at 6 and 12 months after discharge through outpatient clinic visits or telephone contacts. Information was collected regarding adverse events, serious adverse events, MACEs, antiplatelet/antithrombotic medications, and any interventional procedures performed since the last contact.

### 2.9. Endpoints

The primary endpoint was the incidence of in-hospital arrhythmias, including symptomatic sinus bradycardia, Grade II and above atrioventricular block, atrial fibrillation, frequent ventricular premature contractions, ventricular tachycardia, and ventricular fibrillation.

Secondary endpoints included MACEs during the 12-month follow-up period. MACEs were defined as a composite of cardiac death, heart failure, nonfatal myocardial infarction, target vessel/lesion revascularization, and rehospitalization due to cardiac disease.

### 2.10. Outcome Adjudication

All clinical outcomes were independently adjudicated by two experienced cardiologists who were blinded to treatment allocation. Arrhythmias were classified and adjudicated according to the ACC/AHA 2023 arrhythmia classification guidelines [24]. Each arrhythmic event captured by the continuous cardiac monitoring system was independently reviewed by both adjudicators using standardized criteria. In cases of disagreement between the two primary adjudicators, a third independent cardiologist was consulted, and final decisions were reached through consensus discussion among all three reviewers.

### 2.11. Sample Size Calculation

Based on previous literature and preliminary studies by our research group, we estimated that the incidence of in-hospital arrhythmias would be 43% in the control group and 25% in the Shenfu injection group. With a two-sided alpha level of 0.05 and a power of 80% (*β* = 0.2), a sample size of at least 108 patients per group would be required. Considering a potential dropout rate of 10%, we planned to enroll 119 patients per group (at least 238 patients total).

### 2.12. Statistical Analysis

Statistical analyses were performed using SPSS Version 22.0. All tests were two-sided. Continuous variables were presented as mean ± standard deviation. For normally distributed continuous variables, Student's *t*-test or analysis of variance was used; for nonnormally distributed variables, nonparametric tests were applied. Categorical variables were compared using chi-square test or Fisher's exact test as appropriate. Multivariable logistic regression analysis and Kaplan–Meier curves were used to explore risk factors associated with arrhythmias after STEMI. Spearman's correlation analysis was conducted to examine the relationship between infarct size, MVO, and arrhythmia. Both intention-to-treat (ITT) and per-protocol (PP) analyses were performed for the primary endpoint, with results showing consistency between the two analyses. *p* value < 0.05 was considered statistically significant.

### 2.13. Safety and Adverse Events

Safety evaluation was conducted by qualified investigators according to the Technical Guidelines for Causality Assessment of Adverse Events in Clinical Drug Trials (2024 version) [25]. Serious adverse events would be reported to the Institutional Review Board within 24 h. Comprehensive safety monitoring included continuous assessment of vital signs, cardiovascular parameters, and laboratory values throughout the study period.

## 3. Results

### 3.1. Baseline Characteristics

A total of 245 patients were randomized to receive either Shenfu injection (*n* = 123) or control treatment (*n* = 122). The two groups were well balanced across all key demographic and clinical parameters. There was no statistical difference in age, gender, BMI, blood pressure, heart rate, hemoglobin, BNP, or LVEF between the two groups. Cardiovascular risk factors including hypertension, diabetes, and smoking status were comparable. Angiographic and procedural characteristics, including infarct-related artery, vessel disease distribution, Killip classification, symptom-to-first medical contact time, door-to-balloon time, and number of stents implanted were also similar between groups. The majority of patients in both groups received similar rates of guideline-recommended medications including antiplatelets, ACEI/ARB/ARNI, statins, and beta-blockers. All baseline laboratory values were within comparable ranges between the two groups. The detailed baseline characteristics are shown in Table 1.

### 3.2. CMR Imaging

**Figure 2:**
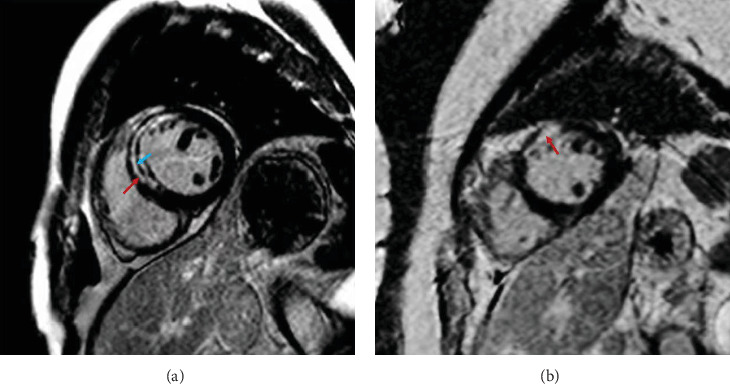
Delayed-enhancement cardiac MRI findings in two patients with acute myocardial infarction caused by occlusion of the left anterior descending coronary artery. (a) MVO in a patient without Shenfu. (b) Another AMI patient treated with Shenfu for 5 days. Only focal endocardial LGE was found. Red arrow: myocardial infarction area. Blue arrow: MVO area of myocardial infarction.

CMR imaging was performed in 174 patients (83 in control group and 91 in Shenfu injection group). Despite comparable baseline cardiac volumes and function, the Shenfu injection group demonstrated significantly smaller infarct size (16.1 ± 9.1 vs. 20.8 ± 13.1 g, *p* = 0.007), representing a 23% reduction in damaged myocardium. Furthermore, MVO—a powerful predictor of adverse outcomes was markedly reduced in the Shenfu group, with both lower incidence (45.0% vs. 65.0%, *p* = 0.008) and smaller extent (2.1 ± 1.5 vs. 3.5 ± 3.3 g, *p* = 0.006) (Table 2). Representative delayed-enhancement cardiac MRI images demonstrate the differential myocardial damage patterns between treatment groups (Figure 2). As illustrated, patients in the control group exhibited extensive MVO areas within the infarct zone (Figure 2a), whereas patients treated with Shenfu injection showed predominantly focal endocardial late gadolinium enhancement with minimal MVO (Figure 2b), consistent with the quantitative measurements showing reduced infarct size and MVO extent. To explore the mechanistic relationship between myocardial damage and arrhythmia occurrence, we performed correlation analysis between CMR parameters and arrhythmia incidence. Spearman's correlation analysis revealed a significant positive correlation between infarct size (as percentage of left ventricular mass) and in-hospital arrhythmia occurrence (*r* = 0.212, *p* = 0.005). Similarly, MVO (as percentage of left ventricular mass) showed a significant correlation with arrhythmia incidence (*r* = 0.229, *p* = 0.026).

### 3.3. Primary Endpoint

Shenfu injection significantly reduced the overall incidence of in-hospital arrhythmias by 37% compared with the control group (24.4% vs. 38.5%, *p* = 0.017). This protective effect was most pronounced for ventricular arrhythmias, with a 53% reduction in frequent ventricular premature contractions (7.3% vs. 15.5%, *p* = 0.042). Although there was no significant difference in life-threatening ventricular tachycardia/fibrillation between the Shenfu injection and control groups, the incidence in the Shenfu injection group was 4.1% lower than in the control group (3.2% vs. 7.3%) (Table 3). To address potential confounding by baseline differences in myocardial injury markers, we performed multivariable logistic regression analysis adjusting for age, three-vessel coronary disease, CK-MB max, TNI max, LVEF, BNP, door-to-balloon time, hypertension, and diabetes mellitus. After this comprehensive adjustment, Shenfu injection remained independently associated with reduced risk of in-hospital arrhythmias (adjusted OR 0.454, 95% CI: 0.249–0.827, *p* = 0.010).

### 3.4. Secondary Outcomes

**Figure 3:**
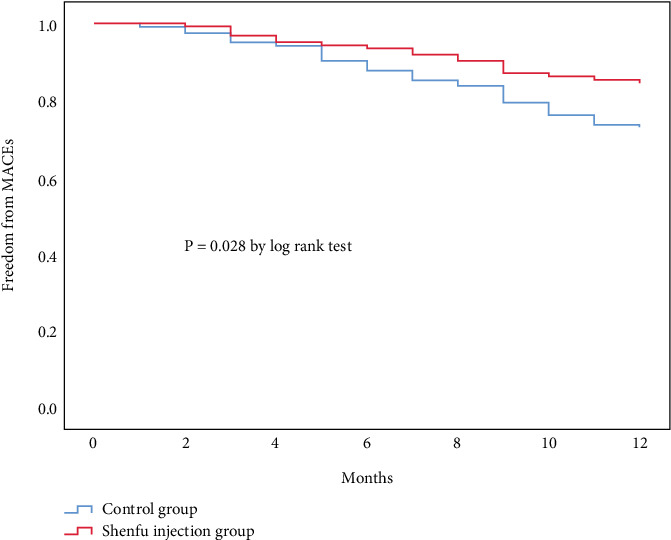
Kaplan–Meier analysis of freedom from major adverse cardiac events.

Most importantly, Shenfu injection significantly reduced MACEs by 43% during follow-up (15.4% vs. 27.0%, *p* = 0.028). Kaplan–Meier analysis confirmed significantly higher freedom from MACEs in the Shenfu injection group compared with the control group (*p* = 0.028 by log-rank test, Figure 3). This substantial clinical benefit was driven by consistent reductions across all components of the composite endpoint, with rehospitalization due to heart disease showing the most notable decrease (7.3% vs. 13.9%, *p* = 0.093), representing a nearly 50% reduction. Although not reaching statistical significance individually, the Shenfu injection group also demonstrated lower rates of cardiac death (2.4% vs. 4.0%) and heart failure (6.5% vs. 9.8%) compared with the control group (Table 4).

### 3.5. Safety Outcomes and Adverse Events

No serious adverse events occurred during the trial period; therefore, no protocol adjustments were necessary. Adverse events were minimal: skin rash (1 case), pruritus (1 case), and mild nausea (3 cases). Only minimal adverse events were reported: one case of skin rash, one case of pruritus, and mild nausea in a small number of patients. All events were transient and resolved without treatment discontinuation, with nausea managed through slow infusion rate adjustment. All adverse events were assessed using standardized criteria, with none classified as “definitely related” or “very likely related” to Shenfu injection [26].

## 4. Discussion

Cardiovascular disease remains a leading cause of mortality worldwide, with an ever-increasing disease burden particularly pronounced in aging populations and developing regions [27, 28]. In this context, our findings that Shenfu injection significantly reduced total arrhythmia events (24.4% vs. 38.5%) and major adverse cardiac events (15.4% vs. 27.0%) in post-PCI STEMI patients carry substantial clinical significance. Notably, the 53% reduction in frequent ventricular premature contractions (7.3% vs. 15.5%, *p* = 0.042) and the trend toward decreased life-threatening ventricular tachycardia/fibrillation (3.2% vs. 7.3%) highlight Shenfu's potent antiarrhythmic properties during the critical postinfarction period when patients are most vulnerable to electrical instability. The observed benefits, attributed to the synergistic actions of ginsenosides and aconitum alkaloids [12], address a critical therapeutic gap in acute cardiac care, where existing antiarrhythmic medications often present challenges of proarrhythmic effects and narrow therapeutic windows.

The mechanistic insights revealed in our study have important implications for personalized medicine approaches in cardiovascular care. The sophisticated ion channel modulation by ginsenosides—activating potassium channels while inhibiting calcium and sodium currents—provides a more physiological approach to arrhythmia management compared to traditional single-channel blockers [29–32]. This multichannel regulation, combined with the *β*-adrenergic modulation by aconitum alkaloids [33, 34], explains the balanced control of both tachyarrhythmias and bradyarrhythmias observed in our trial, which may be particularly beneficial in elderly patients and those with structural heart disease where conventional antiarrhythmic drugs often pose increased risks. Importantly, our study provides compelling evidence that these mechanistic effects translate directly into measurable clinical benefits. Our correlation analysis demonstrates significant positive correlations between infarct size and MVO with arrhythmia occurrence, establishing a clear mechanistic pathway from myocardial protection to arrhythmia prevention. These mechanistic insights are substantiated by our cardiac MRI findings, which revealed significantly reduced infarct size (representing a 23% reduction) and lower MVO incidence (45.0% vs. 65.0%) in the Shenfu injection group. This mechanistic coherence, where reduced tissue damage directly translates to decreased electrical instability, strengthens the biological plausibility of our findings and supports the therapeutic rationale for Shenfu injection in acute myocardial infarction. The observed myocardial protection is particularly significant given that despite advances in primary PCI techniques, MVO remains a significant challenge associated with poor long-term outcomes [35, 36]. The ability of Shenfu injection to address this “final frontier” in myocardial reperfusion through multiple pathways—including enhanced mitochondrial function, reduced oxidative stress, and attenuated inflammation [20, 37, 38]—represents a significant advance in comprehensive myocardial protection strategies.

The molecular mechanisms underlying these benefits highlight the potential for integrating traditional medicine with precision cardiology [39]. Modern pharmacological studies revealing ginsenosides' effects on prostaglandin release, coronary vasodilation, and cAMP/cGMP ratio optimization suggest potential synergies with existing pharmacological therapies [29, 40]. This is particularly relevant as healthcare systems increasingly emphasize evidence-based integration of traditional and modern approaches [41], as outlined in both the WHO Traditional Medicine Strategy 2024–2034 and various national healthcare initiatives.

In the current era of value-based healthcare, our findings have significant economic implications. The reduction in hospital stays and rescue interventions, coupled with improved clinical outcomes, addresses the growing need for cost-effective therapeutic strategies. This is especially pertinent in the postpandemic healthcare landscape, where systems worldwide face unprecedented pressure to optimize resource utilization while maintaining quality of care [42, 43]. The comprehensive cardioprotective effects observed could potentially reduce the burden of post-MI complications and associated healthcare costs.

However, critical limitations must be addressed. This was an open-label, unblinded study design, which may introduce observer bias in outcome assessment and potentially influence both patient and physician behavior, despite our use of objective monitoring systems and standardized adjudication protocols. Although our single-center trial demonstrated promising results, multicenter studies are needed to validate these findings across different healthcare settings and patient populations, with larger sample sizes and longer term follow-up periods to better assess the durability of benefits and capture rare but clinically important adverse events. The optimal timing and duration of Shenfu injection administration require further investigation, particularly in relation to PCI timing and other concurrent therapies, whereas pharmacogenomic studies could help identify patient subgroups most likely to benefit from this intervention, aligning with the principles of precision medicine. Future research should prioritize several key areas: large-scale multicenter trials incorporating health economic analyses and long-term follow-up data, studies exploring optimal therapeutic regimens and potential drug interactions particularly with novel antithrombotic and anti-inflammatory agents, investigation of specific molecular pathways using advanced proteomics and metabolomics approaches to further elucidate mechanisms of action, development of biomarker-guided treatment strategies to optimize patient selection, and real-world studies focusing on special populations such as elderly patients, diabetics, and those with chronic kidney disease.

In conclusion, our study provides robust evidence supporting Shenfu injection's integration into contemporary cardiovascular care, particularly in the setting of acute myocardial infarction. The demonstrated benefits, underpinned by sophisticated molecular mechanisms and validated by advanced imaging, represent a successful translation of traditional medicine into evidence-based practice. As healthcare systems worldwide seek sustainable solutions to manage the growing burden of cardiovascular disease, this comprehensive therapeutic approach, characterized by multiple complementary mechanisms of action, demonstrated efficacy, and a favorable safety profile, offers a promising path forward in the evolution of cardiovascular medicine.

## Figures and Tables

**Table 1:** Baseline and procedural characteristics of study participants.

	**Control, ** **n** = 122	**Shenfu injection, ** **n** = 123	**p**
Age (years)	64.6 ± 12.0	64.0 ± 14.0	0.746
Male (gender)	100 (81.9%)	98 (79.6%)	0.649
BMI (kg/m^2^)	24.5 ± 3.3	25.1 ± 6.0	0.300
SBP (mmHg)	124 ± 19	126 ± 16	0.570
DBP (kg/m^2^)	78 ± 12	78 ± 13	0.988
Heart rate (BPM)	83.9 ± 16.4	84.8 ± 15.2	0.688
Hemoglobin (g/L)	137.8 ± 17.7	139.6 ± 16.1	0.412
BNP (pg/mL)	208.6 ± 486.1	221.2 ± 365.2	0.819
CK-MB max (u/L)	353 ± 198	296 ± 163	0.015
TNI max (ng/mL)	58.8 ± 29.2	48.9 ± 26.7	0.006
LDL-C	3.3,0.8	3.3,1.0	0.919
Serum K	3.8,0.4	3.7,0.4	0.090
Hypertension	75 (61.4%)	84 (68.2%)	0.264
Diabetes	47 (38.5%)	45 (36.5%)	0.754
Smoking	58 (47.5%)	60 (48.7%)	0.846
Creatinine	76.5 + 22.3	80.7 + 21.8	0.135
LVEF	51.3,7.8	50.8,8.1	0.585
Angiographic characteristics			
Infarct-related artery			0.260
LAD	57 (46.7%)	67 (54.5%)	
LCX	21 (17.2%)	13 (10.6%)	
RCA	44 (36.1%)	43 (35.0%)	
Coronary lesion			
One-vessel	62 (50.8%)	59 (48.0%)	0.655
Two-vessel	45 (36.9%)	44 (35.8%)	0.856
Three-vessel	15 (12.3%)	20 (16.3%)	0.375
Killip classification			
I/II/III/IV	86/30/6/0	80/34/9/0	0.588
TIMI before PCI			
0/1/2/3	94/6/10/12	99/7/7/10	0.822
Symptom to FMC (h)	4.9 ± 2.9	5.1 ± 2.2	0.492
D to B (min)	69 ± 18	67 ± 15	0.385
PCI characteristics and medications			
Number of stent implanted	1.19 ± 0.43	1.20 ± 0.46	0.797
Thrombus aspiration	26 (21.3%)	30 (24.4%)	0.566
Tirofiban	90 (73.8%)	83 (67.5%)	0.280
Aspirin	116 (95.1%)	115 (93.5%)	0.593
Clopidogrel	15 (12.3%)	11 (8.9%)	0.394
Ticargrelor	107 (87.7%)	112 (91.1%)	0.394
ACEI/ARB/ARNI	104 (85.2%)	102 (82.9%)	0.620
Statins	109 (89.3%)	110 (89.4%)	0.982
Beta-blockers	97 (79.5%)	96 (78.0%)	0.780
Oral anticoagulation	5 (4.1%)	6 (4.9%)	0.768
Thrombus aspiration	26 (21.3%)	30 (24.4%)	0.566

*Note:* Data are presented as mean ± SD or number (%) of subjects.

Abbreviations: ACEI/ARB/ARNI, angiotensin-converting enzyme inhibitor/angiotensin II receptor blocker/angiotensin receptor-neprilysin inhibitor; BMI, body mass index; BNP, brain natriuretic peptide; BPM, beats per minute; DBP, diastolic blood pressure; D to B, door to balloon; FMC, first medical contact; K, potassium; LAD, left anterior descending; LCX, left circumflex; LDL-C, low-density lipoprotein cholesterol; LVEF, left ventricular ejection fraction; RCA, right coronary artery; SBP, systolic blood pressure.

**Table 2:** Cardiac magnetic resonance imaging assessment.

	**Control, ** **n** = 83	**Shenfu injection, ** **n** = 91	**p**
LVEDV, mL	148.9 ± 25.6	148.4 ± 26.0	0.896
LVESV, mL	79.1 ± 17.3	77.4v17.1	0.518
LVEF, %	46.9 ± 7.4	47.5 ± 8.3	0.602
LVmass, g	113.8 ± 18.9	113.0 ± 35.3	0.848
Infarct size, g	20.8 ± 13.1	16.1 ± 9.1	0.007
Infarct/LV mass, %	17.8 ± 10.7	14.4 ± 7.6	0.018
MVO, %	54 (65.0%)	41 (45.0%)	0.008
MVO, g	3.5 ± 3.3	2.1 ± 1.5	0.006
MVO/LV mass, %	2.95 ± 2.79	1.88 ± 1.43	0.017
MVO/infarct size, %	15.27 ± 12.94	10.15 ± 6.95	0.015

*Note:* Data are presented as mean ± SD, median (interquartile range), or number (%) of subjects.

Abbreviations: LV, left ventricular; LVEDV, left ventricular end-diastolic volume; LVEF, left ventricular ejection fraction; LVESV, left ventricular end-systolic volume; MVO, microvascular obstruction.

**Table 3:** Incidence of in-hospital arrhythmia events.

	**Control, ** **n** = 122	**Shenfu injection, ** **n** = 123	**p**
Total arrhythmia events	47 (38.5%)	30 (24.4%)	0.017
Symptomatic sinus bradycardia or Grades II and III AVB	21 (17.2%)	14 (11.3)	0.192
Atrial fibrillation	10 (8.1%)	8 (6.5%)	0.612
Frequent ventricular premature	19 (15.5%)	9 (7.3%)	0.042
Ventricular tachycardia and ventricular fibrillation	9 (7.3%)	4 (3.2)	0.150

**Table 4:** Clinical outcomes during the 12-month follow-up.

	**Control, ** **n** = 122	**Shenfu injection, ** **n** = 123	**p**
Major adverse cardiac events	33 (27.0%)	19 (15.4%)	0.028
Cardiac death	5 (4.0%)	3 (2.4%)	0.500
Heart failure	12 (9.8%)	8 (6.5%)	0.341
Nonfatal myocardial infarction	2 (1.6%)	1 (0.8%)	0.622
Target vessel/lesion revascularization	2 (1.6%)	1 (0.8%)	0.622
Rehospitalization due to heart disease	17 (13.9%)	9 (7.3%)	0.093

## Data Availability

The data that support the findings of this study are available from the corresponding author upon reasonable request.

## References

[1] Thomsen A. F., Jacobsen P. K., Køber L. (2021). Risk of Arrhythmias After Myocardial Infarction in Patients With Left Ventricular Systolic Dysfunction According to Mode Of Revascularization: A Cardiac Arrhythmias and Risk Stratification After Myocardial Infarction (CARISMA) Substudy. *Europace*.

[2] Byrne R. A., Rossello X., Coughlan J. J. (2023). 2023 ESC Guidelines for the Management of Acute Coronary Syndromes. *European Heart Journal*.

[3] Byrne R. A., Rossello X., Coughlan J. J. (2024). Correction to: 2023 ESC Guidelines for the Management of Acute Coronary Syndromes: Developed by the Task Force on the Management of Acute Coronary Syndromes of the European Society of Cardiology (ESC). *European Heart Journal*.

[4] Clements-Jewery H., Hearse D. J., Curtis M. J. (2005). Phase 2 Ventricular Arrhythmias in Acute Myocardial Infarction: A Neglected Target for Therapeutic Antiarrhythmic Drug Development and for Safety Pharmacology Evaluation. *British Journal of Pharmacology*.

[5] Sandoval Y., Jaffe A. S. (2019). Type 2 Myocardial Infarction. *Journal of the American College of Cardiology*.

[6] Chan W., Duffy S. J., White D. A. (2012). Acute Left Ventricular Remodeling Following Myocardial Infarction: Coupling of Regional Healing With Remote Extracellular Matrix Expansion. *JACC: Cardiovascular Imaging*.

[7] Winkler C., Funk M., Schindler D. M., Hemsey J. Z., Lampert R., Drew B. J. (2013). Arrhythmias in Patients With Acute Coronary Syndrome in the First 24 Hours of Hospitalization. *Heart & Lung*.

[8] Xu X., Wang Z., Yang J., Fan X., Yang Y. (2024). Burden of Cardiac Arrhythmias in Patients With Acute Myocardial Infarction and Their Impact on Hospitalization Outcomes: Insights From China Acute Myocardial Infarction (CAMI) Registry. *BMC Cardiovascular Disorders*.

[9] Rowlands D. J. (1978). The Management Of Arrhythmias Following an Acute Myocardial Infarction. *Intensive Care Medicine*.

[10] Steg P. G., James S. K., Atar D. (2012). ESC Guidelines for the Management of Acute Myocardial Infarction in Patients Presenting With ST-Segment Elevation. *European Heart Journal*.

[11] Priori S. G., Blomström-Lundqvist C., Mazzanti A. (2015). 2015 ESC Guidelines for the Management of Patients With Ventricular Arrhythmias and the Prevention of Sudden Cardiac Death: The Task Force for the Management of Patients With Ventricular Arrhythmias and the Prevention of Sudden Cardiac Death of the European Society of Cardiology (ESC). Endorsed by: Association for European Paediatric and Congenital Cardiology (AEPC). *European Heart Journal*.

[12] Xu F.-F., Xie X.-F., Hu H.-Y., Tong R.-S., Peng C. (2024). Shenfu Injection: A Review of Pharmacological Effects on Cardiovascular Diseases. *Frontiers in Pharmacology*.

[13] Wang X., Miao H., Yan Y. (2021). Effect of Shenfu Injection on Reperfusion Injury in Patients Undergoing Primary Percutaneous Coronary Intervention for ST Segment Elevation Myocardial Infarction: A Pilot Randomized Clinical Trial. *Frontiers in Cardiovascular Medicine*.

[14] Tao L., Mo Z., Li Z. (2023). Efficacy and Safety of Shenfu Injection on Acute Heart Failure: A Systematic Review and Meta-Analysis. *Phytomedicine*.

[15] Wu Y., Li S., Li Z. (2022). Efficacy and Safety of Shenfu Injection for the Treatment of Post-Acute Myocardial Infarction Heart Failure: A Systematic Review and Meta-Analysis. *Frontiers in Pharmacology*.

[16] Xu P., Zhang W.-Q., Xie J., Wen Y.-S., Zhang G.-X., Lu S.-Q. (2020). Shenfu Injection Prevents Sepsis-Induced Myocardial Injury by Inhibiting Mitochondrial Apoptosis. *Journal of Ethnopharmacology*.

[17] Li L., Ye J., Zhao Z. (2024). Shenfu Injection Improves Isoproterenol-Induced Heart Failure in Rats by Modulating Co-Metabolism and Regulating the trimethylamine-N-Oxide - Inflammation Axis. *Frontiers in Pharmacology*.

[18] Chen R.-J., Rui Q.-L., Wang Q., Tian F., Wu J., Kong X.-Q. (2020). Shenfu Injection Attenuates Lipopolysaccharide-Induced Myocardial Inflammation and Apoptosis in Rats. *Chinese Journal of Natural Medicines*.

[19] Huang P., Guo Y., Hu X., Fang X., Xu X., Liu Q. (2024). Mechanism of Shenfu Injection in Suppressing Inflammation and Preventing Sepsis-Induced Apoptosis in Murine Cardiomyocytes Based on Network Pharmacology and Experimental Validation. *Journal of Ethnopharmacology*.

[20] Yuan W., Wu J.-Y., Wang G.-X., Zhang Q., Li C.-S. (2015). Effect of Shen-Fu Injection Pretreatment to Myocardial Metabolism During Untreated Ventricular Fibrillation in a Porcine Model. *Chinese Medical Journal*.

[21] Gu W., Li C., Yin W., Guo Z., Hou X., Zhang D. (2012). Shen-fu Injection Reduces Postresuscitation Myocardial Dysfunction in a Porcine Model of Cardiac Arrest by Modulating Apoptosis. *Shock*.

[22] Ji X.-F., Ji H.-B., Sang D.-Y., Wang S., Yang L., Li C.-S. (2013). Shen-Fu Injection Reduces Impaired Myocardial *β*-Adrenergic Receptor Signaling After Cardiopulmonary Resuscitation. *Chinese Medical Journal*.

[23] Rao S. V., O’Donoghue M. L., Ruel M. (2025). Correction to: 2025 ACC/AHA/ACEP/NAEMSP/SCAI Guideline for the Management of Patients With Acute Coronary Syndromes: A Report of the American College of Cardiology/American Heart Association Joint Committee on Clinical Practice Guidelines. *Circulation*.

[24] Joglar J. A., Chung M. K., Armbruster A. L. (2024). 2023 ACC/AHA/ACCP/HRS Guideline for the Diagnosis and Management of Atrial Fibrillation: A Report of the American College of Cardiology/American Heart Association Joint Committee on Clinical Practice Guidelines. *Circulation*.

[26] Zhao X. (2018). Expert Consensus on Clinical Application of Shenfu Injection in Acute and Severe Cases. *Journal of Clinical Emergency (China)*.

[27] Goldsborough E., Osuji N., Blaha M. J. (2022). Assessment of Cardiovascular Disease Risk. *Endocrinology and Metabolism Clinics of North America*.

[28] Leong D. P., Joseph P. G., McKee M. (2017). Reducing the Global Burden of Cardiovascular Disease, Part 2: Prevention and Treatment of Cardiovascular Disease. *Circulation Research*.

[29] Fan W., Huang Y., Zheng H. (2020). Ginsenosides for the Treatment of Metabolic Syndrome and Cardiovascular Diseases: Pharmacology and Mechanisms. *Biomedicine & Pharmacotherapy*.

[30] Sarhene M., Ni J. Y., Duncan E. S. (2021). Ginsenosides for Cardiovascular Diseases; Update on Pre-Clinical and Clinical Evidence, Pharmacological Effects and the Mechanisms of Action. *Pharmacological Research*.

[31] Ramli F. F., Ali A., Ibrahim N. I. (2022). Molecular-Signaling Pathways of Ginsenosides Rb in Myocardial Ischemia-Reperfusion Injury: A Mini Review. *International Journal of Medical Sciences*.

[32] Sun Y., Liu Y., Chen K. (2016). Roles and Mechanisms of Ginsenoside in Cardiovascular Diseases: Progress and Perspectives. *Science China Life Sciences*.

[33] Yang L., Xie G., Wang Y. (2022). Metabolic Behaviors of Aconitum Alkaloids in Different Concentrations of Aconiti Lateralis Radix Praeparata and Effects of Aconitine in Healthy Human and Long QT Syndrome Cardiomyocytes. *Molecules*.

[34] Xing B.-N., Jin S.-S., Wang H. (2014). New Diterpenoid Alkaloids From *Aconitum coreanum* and Their Anti-Arrhythmic Effects on Cardiac Sodium Current. *Fitoterapia*.

[35] Niccoli G., Scalone G., Lerman A., Crea F. (2016). Coronary Microvascular Obstruction in Acute Myocardial Infarction. *European Heart Journal*.

[36] Vora K. P., Kumar A., Krishnam M. S., Prato F. S., Raman S. V., Dharmakumar R. (2024). Microvascular Obstruction and Intramyocardial Hemorrhage in Reperfused Myocardial Infarctions: Pathophysiology and Clinical Insights From Imaging. *JACC: Cardiovascular Imaging*.

[37] Guo Z.-J., Wu C.-J., Li C.-S. (2016). Shen-Fu Injection Alleviates Post-Resuscitation Myocardial Dysfunction by Up-Regulating Expression of Sarcoplasmic Reticulum Ca^2+^-ATPase. *Chinese Journal of Integrative Medicine*.

[38] Pan C., Li J., Han G., Xu T., Li M. (2019). Effects of Shen-Fu Injection on Mitochondrial Function in the Intestinal Epithelial Cells of Rats With Endotoxemia. *Pakistan Journal of Pharmaceutical Sciences*.

[39] Zhang P., Zhang D., Zhou W. (2023). Network Pharmacology: Towards the Artificial Intelligence-Based Precision Traditional Chinese Medicine. *Briefings in Bioinformatics*.

[40] Liu L., Jin M., Han X., Dou D. (2024). Identifying Biomarkers of Ginseng Medicines With Different Natures on Heart Failure. *Journal of Ethnopharmacology*.

[41] Kendi N. N. (2024). Impact of Traditional Medicine Integration with Modern Healthcare in Africa. *Newport International Journal of Scientific and Experimental Sciences (NIJSES)*.

[42] Doarn C. R. (2021). The Changing Landscape of Health Care. *Telemedicine Journal and E-Health*.

[43] Lewis K. O., Popov V., Fatima S. S. (2024). From Static Web to Metaverse: Reinventing Medical Education in the Post-Pandemic Era. *Annals of Medicine*.

